# Digital Transformation and Open Innovation Planning of Response to COVID-19 Outbreak: A Systematic Literature Review and Future Research Agenda

**DOI:** 10.3390/ijerph20032731

**Published:** 2023-02-03

**Authors:** Ben Zhang, Chenxu Ming

**Affiliations:** 1 Law School, Huazhong University of Science and Technology, Wuhan 430074, China; 2 Sino-European Institute for Intellectual Property, Huazhong University of Science and Technology, Wuhan 430074, China; 3 School of Management, Huazhong University of Science and Technology, Wuhan 430074, China

**Keywords:** digital transformation, bibliometric analysis, COVID-19 pandemic, open innovation system, epidemic prevention and control, public health governance

## Abstract

The COVID-19 pandemic highlights the importance of digital technology in a specific region’s epidemic prevention and control, and the digital transformation strategy based on the open innovation system is an emerging way to tackle conceivable outbreaks. Based on the bibliometric study of relevant literature data, this paper evaluated the research and development status in this field, and conducted a systematic literature review on the basis of the core articles identified. The results of bibliometric analysis software, including CiteSpace, CitNetExplorer and VOSViewer, showed that the development of relevant research presented rapidity and decentralization, and the evolution process of literature topics further implies the necessity of interdisciplinary and multisectoral collaboration. Furthermore, this paper summarized the specific implementation strategies for constructing an open innovation system, and discussed the role and development plan of digital technology in epidemic prevention and control.

## 1. Introduction

During the three-year fight against the coronavirus disease 2019 (COVID-19) pandemic, a variety of digital technology applications have emerged to achieve public health governance, such as the Internet, artificial intelligence and blockchain [1]. Considering the need for epidemic prevention and control in a region, digital technology can reflect the real-time situation of an outbreak, and predict the trend of epidemics based on mathematical models and a large number of calculations, thus helping the government make timely response decisions. From the perspective of economic development, digital technology is an effective means to achieve the resumption of work and production under the premise of ensuring personal health. Through tracing the health status of employees in a timely manner, enterprise operators can quickly deploy work arrangements in the information system, and enable employees in isolation to participate in work. Digital technology has provided advantages in responding to the COVID-19 pandemic, and there is still great potential to be tapped. Moreover, the severe virus variant outbreak has forced digitalization for specific regions. In terms of the impact of the new virus, the traditional prevention methods have shown weaknesses. Introducing digital technology applications is urgent to accelerate the transformation of epidemic blockade strategies.

However, the most important challenge faced by digital technology applications is the greater uncertainty in the process of epidemic prevention. Due to the rapid development of technological innovation and the diversification of technological applications, the use of digital technology presents the characteristic of fragmentation. The lack of systematic management and planning for epidemic prevention will limit the positive effects of digital technology, and may also lead to personal information violations or other negative effects. Some studies have conducted bibliometric research on the COVID-19 pandemic [2,3,4], and the discussion indicates that digital transformation needs more attention. The bibliometric analysis of the COVID-19 pandemic situation and digital transformation can provide deep insights. Therefore, the design of this study aims to fully combine the development status of epidemic prevention and digital technology on the basis of some innovation theories, such as open innovation and knowledge sharing [5]. Some studies [6,7,8] have provided a theoretical basis for implementing digital transformation in the context of open innovation, but the relations to the COVID-19 pandemic do not seem to be enough. Open innovation refers to a peculiar innovation paradigm formed by breaking through organizational boundaries and utilizing external resources beyond the organization [9]. The government or other public institutions are the main policy implementers in epidemic prevention, but the implementation process also relies on the joint participation of enterprises, the public and other external entities. Therefore, an effective open innovation mechanism can unite and coordinate various entities to play their roles, thus curbing epidemic development. In any case, constructing the open innovation mechanism is an important response strategy and also a public health governance tool inspired by prior research.

Based on the above background, this paper proposes a research question: how to systematically apply digital technology to support the prevention and control strategy for the COVID-19 pandemic? In view of the above main problems, this study intends to use bibliometric research methods to carry out an analysis. Through collecting relevant articles in the field as analysis samples, this study uses CiteSpace, CitNetExplorer and VOSViewer to explore the literature data, thus identifying some key articles for systematic review. Furthermore, we analyze the thematic and citation relationships between the key articles, proposing systematic planning to build an open innovation mechanism with digital technology for fighting against the COVID-19 pandemic. The bibliometric analysis and systematic literature review focus the scattered literature data into a system framework, which is helpful to conclude diversified schemes of digital technology application.

## 2. Research Design

### 2.1. Data Source and Process

This study used the literature data for bibliometric analysis and systematic literature review analysis. The literature data were obtained from the Web of Science core database. (https://www.webofscience.com, accessed on 1 July 2022). The retrieval date was 7 November 2022. In terms of setting the scope of the literature, the citation included all indexes, and the literature type was the article. Finally, 11,377 documents were retrieved. The established retrieval strategy is as follows:

(TS = (COVID-19) OR TS = (coronavirus 2) OR TS = (2019 novel coronavirus disease) OR TS = (SARS-CoV-2) OR TS = (novel coronavirus) OR TS = (2019-nCoV) OR TS = (coronavirus disease 2019) OR TS = (coronavirus disease-19)) AND (TS = (digital transformation) OR TS = (digital technology) OR TS = (information technology) OR TS = (Internet technology) OR TS = (artificial intelligence) OR TS = (robot) OR TS = (blockchain) OR TS = (cloud computing) OR TS = (virtual reality) OR TS = (augmented reality) OR TS = (Internet of Things) OR TS = (Web of Things) OR TS = (IoT) OR TS = (Internet plus) OR TS = (intelligent manufacturing) OR TS = (smart manufacturing) OR TS = (cyberspace) OR TS = (network space) OR TS = (network security) OR TS = (Internet security) OR TS = (cyber security) OR TS = (Metaverse))

The above retrieval strategy can be used in the advanced retrieval function of the WOS database. In addition, with the development of relevant research, the subsequent relevant research can revise the search strategy for specific topics. The basic idea of this search strategy is to combine the content related to the COVID-19 pandemic and digital transformation, and we hope to find a connection between the two topics. Furthermore, using the data processing function and analytical indicators of bibliometrics can focus the research perspective on influential documents. Therefore, the cleaning of the raw data is included in the bibliometric analysis.

In addition, this study designed a criterion for literature selection. On one side, the literature related to the retrieval topic is retained. On the other side, the superabundant literature is excluded based on the bibliometric analysis, since this study aimed to explore the retrieval according to the core studies. Moreover, the exclusion criteria are based on the bibliometric performance, which is set in the specific bibliometric analysis as shown in Section 3.2. This study constructed a keyword co-occurrence network and a literature co-citation network, and some network indicators were used to be the exclusion criteria, such as g-index, link retaining factor (LRF), maximum links per node (L/N) and look back years (LBY), etc.

### 2.2. Analysis Process

This paper used the bibliometric software Citespace, CitNetExplorer and VOSviewer to conduct literature text mining analysis. Citespace (https://citespace.podia.com/, accessed on 1 November 2022) contains many analysis modules, and the representative analysis functions include finding turning points in the evolution trend [10,11], visual analysis [12,13], co-citation analysis and cluster analysis [14] for a specific research field. In addition, CitNetExplorer and VOSviewer are suitable for analyzing small-scale datasets. CitNetExplorer (https://www.citnetexplorer.nl/, accessed on 1 November 2022) is mainly used for citation network analysis [15] and can visually show the dynamic evolution for a specific research field over time. VOSviewer (https://www.vosviewer.com/, accessed on 1 November 2022) can visualize the network structure of literature from different dimensions [16,17] and highlight the most prominent part.

Since the main purpose was to find digital technology applications directly related to combating COVID-19 from a large number articles, using the method of text mining for literature analysis was necessary. Moreover, the bibliometric methods can find the most valuable documents from more than 10,000 documents, greatly improving the efficiency of document collation and systematic analysis. As shown in Table 1, the analysis mainly included the following three steps:

Step 1: Overall distribution analysis. This step analyzes the quantity distribution characteristics of relevant literature from multiple descriptive dimensions, including basic bibliometric distribution, research content distribution, research geography and foundation. Moreover, the WOS database is used for preliminary analysis of literature data, thus helping to understand the evolution process in the research field.

Step 2: Literature network structure analysis. On the basis of the literature data, this step constructed the keyword co-occurrence network and literature co-citation network, and performed the text clustering and other methods to identify documents with high importance. This paper mainly selects the modules of keyword co-occurrence network analysis and co-citation network analysis through Citespace. Keywords usually represent the most frequently used content in the literature, thus the co-occurrence relationship between keywords can reflect the semantic structure of the research field, and further indicates the dynamic development trend over time. Co-citation usually reflects the relationship between two cited documents, and the resulting knowledge map shows the most closely related parts of the literature collection in a large number of documents. Based on the analysis rule, we can identify the aforementioned specific literature collection through the frequency of co-citation, thus finding the knowledge base and research front. Furthermore, CitNetExplorer and VOSviewer are used to identify core literature and verify the analysis results of Citespace software. CitNetExplorer illustrates the citation evolution path and VOSviewer further clusters the focused topics. By tracing the literature data contained in each clustering topic, the core articles to be analyzed are identified.

Step 3: Content analysis of core literature. According to the identified core documents, this step divides the specific text content of the documents into different subject categories, and then the analysis focuses on the content of digital technology applications.

## 3. Literature Bibliometric Analysis

### 3.1. Overall Distribution Analysis

#### 3.1.1. Basic Bibliometric Distribution

**Figure 1:**
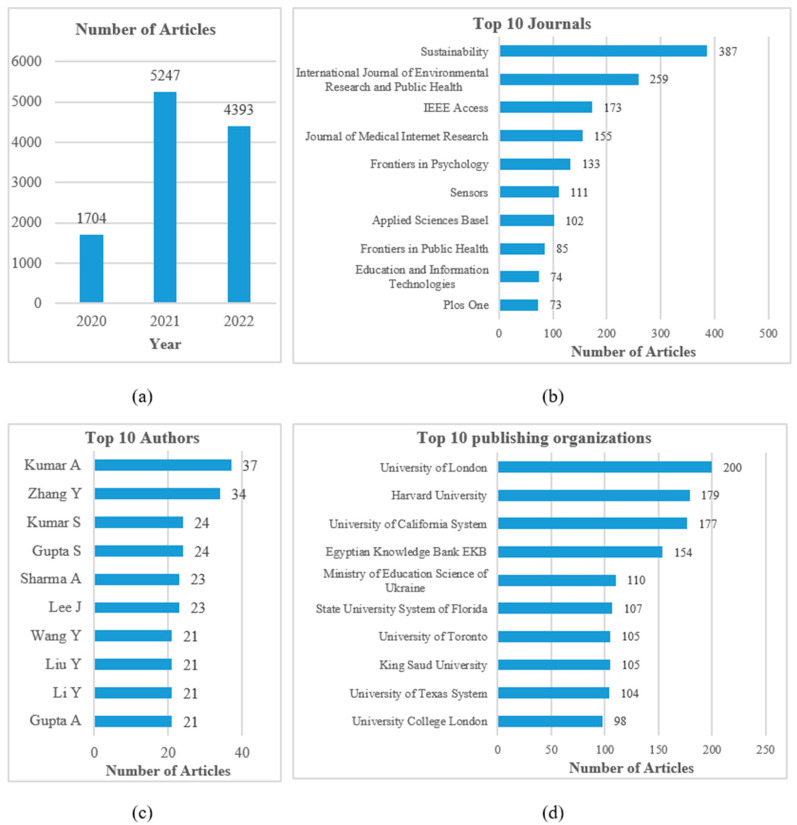
Literature distribution in four basic dimensions: (**a**) the article quantities by year; (**b**) the top 10 journals; (**c**) the top 10 authors; (**d**) the top 10 publishing organizations.

Figure 1 reflects four aspects of literature distribution. The four subgraphs in the figure, respectively, reflect the article quantities by year, the distribution of the top 10 journals publishing related articles, the distribution of the top 10 authors and the distribution of the top 10 publishing organizations. Since COVID-19 began to break out in 2020, the corresponding Figure 1a shows that relevant research is mainly distributed in the time interval of 2020, 2021 and 2022. The number in 2021 increases by three times compared with that in 2020. The number in 2022 slows down to a certain extent, but the overall number is still at a high level. Figure 1b shows that the main journals are from various disciplines, which shows that the digitalization of epidemic prevention and control is an interdisciplinary topic. Figure 1c reflects the fact that many scholars have rapidly reached a large number of achievements in the research field. Figure 1d presents the main publishing organizations, such as the University of London, Harvard University and the University of California System, which have the most research outputs.

From the presented distribution information, the relevant research on digital transformation in fighting against the pandemic has received extensive attention and is continuing to develop rapidly. However, Figure 1 only shows the basic research evolution process and macro element distribution information in the research field. In order to further explore the core issues in this field and predict future research trends, this study next analyses the deep contents by using some bibliometric tools.

#### 3.1.2. Research Topic Distribution

Table 2 shows three literature classification methods, including research areas, WOS categories and citation topics meso. The table lists the top 10 categories and the corresponding literature number for each classification method. Research areas reflect the main disciplines related to the analyzed literature, while the WOS categories summarize the themes around which the literature forms clusters. Citation topics is a new and dynamic document classification method provided by the WOS database, exploring the topic evolution in a certain period and further showing the classification structure at the middle literature level. In a word, these three classification methods show the internal theme structure of the literature dataset from each perspective, and can also provide reliable verification for the core theme identification. For example, computer science and engineering are the two topic categories that rank first and second in the research areas. However, in WOS categories, categories similar to the above topics are ranked lower. For example, the category of computer science information systems ranks second, engineering electrical electronic ranks sixth and education educational research ranks first. The distribution of categories in citation topics meso is similar, and the corresponding article number to each category is significantly reduced compared with research areas due to the further refinement of topics.

Furthermore, Table 2 shows that the digital transformation of epidemic prevention and control is also a very important social issue aside from the technical content. Due to the rapid spread of the virus and the long incubation period, epidemic prevention is facing a serious and complex situation. In particular, for the new variant of Omicron, the transmission has a faster speed, stronger infectivity and shorter intergenerational interval in the population. Therefore, regional health departments need to adjust the corresponding public health policies and epidemic prevention according to specific local conditions. Although there is a great dispute about the best prevention and control model, a clear consensus is to strengthen the role of digital technology in epidemic prevention and control. Considering the current situation, all sectors of society urgently need a feasible plan to carry out relevant actions for digital transformation. Furthermore, digitalization is the key to contacting various industrial clusters to jointly control the epidemic. Through the collaboration and standardization of various departments on digital technology, the government can achieve the outbreak prevention goals more accurately, faster and earlier.

#### 3.1.3. Research Geography and Foundation

**Figure 2:**
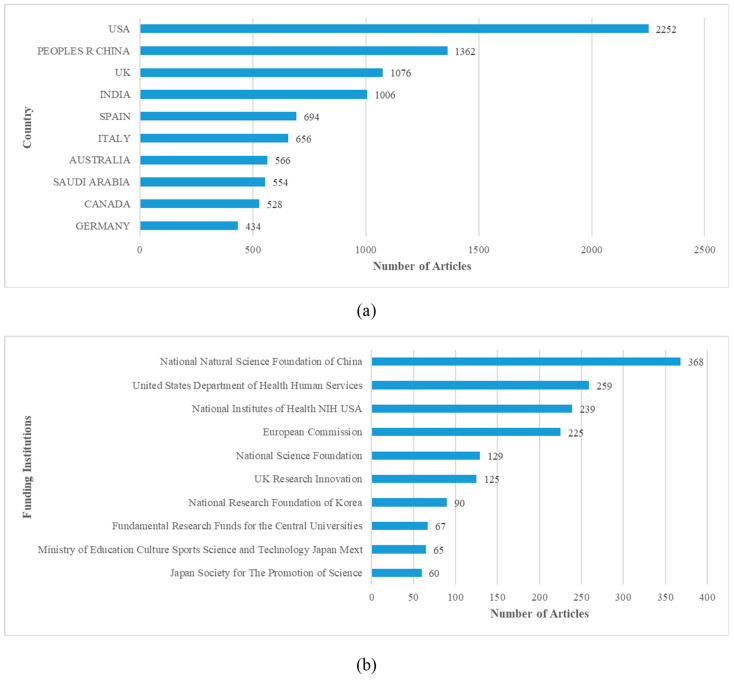
Top 10 literature sources of (**a**) countries/regions and (**b**) funding institutions.

Figure 2 shows the literature distribution of countries/regions and funding institutions. The statistics show that there are 161 countries or regions of research origin and 5998 funding institutions. In general, this research field has received extensive attention worldwide. Among the sub-graphs, Figure 2a shows the top 10 geographic sources in terms of article quantity, with the United States, China and the United Kingdom ranking in the top three. Figure 2b shows the top 10 sources of funding institutions, with the National Natural Science Foundation of China supporting the largest number of articles, followed by the United States Department of Health Human Services and the National Institutes of Health USA. As can be seen from Figure 2, the United States is the most important source of research output, and the research quantity shows that the relevant funding support has played a good role. The number of research papers in China ranks second, which is only next to that in the United States, but the funding institution number is the largest, which also contributes a large proportion of research output to this research field.

### 3.2. Literature Network Structure Analysis

#### 3.2.1. Keyword Co-Occurrence Network Analysis

In the keyword analysis process of Citespace, no part of the literature data is included in the analysis scope. Although 11,377 pieces of data were imported into Citespace software, there are only 10,569 pieces identified in the period 2020–2022. In addition, the amount of data is so large that the core keywords are submerged. Therefore, the analysis process needs a screening standard to narrow the analysis sample, then the analysis perspective can focus on the evolution of the core keywords. As shown in Table 3, the g-index is used to select keywords, which means the number of citations obtained is no less than the square of the g value for a researcher’s first g published papers (ranked according to the number of citations). The number of keywords obtained from the last three 1-year slices is 272, 586 and 599, respectively, and the corresponding number of connections related to these keywords is 816, 1758 and 1797, respectively. The k value in the table is a scaling factor and a parameter in the g-index. If the analysis process needs to obtain larger analysis samples, the keyword network can add more nodes by increasing the k value.

**Figure 3:**
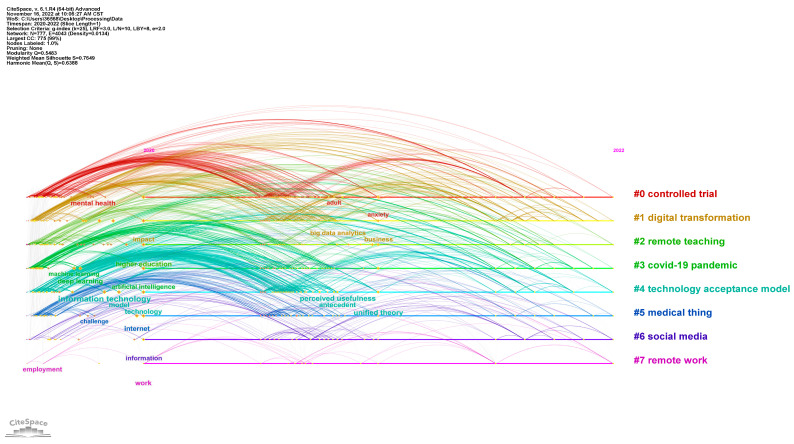
Keyword co-occurrence network (timeline view). This figure omits the labels of some nodes avoid congestion, which highlights the network links. The colors of links correspond to the categories. The label of a node is the keyword extracted from the article title. The horizontal axis is the publication time, and the vertical axis shows the main clustering categories.

As mentioned above, due to a large number of keywords, this paper only analyzes the core part of the raw data and simplifies the network structure. The network node screening criteria include the g-index (k = 25), LRF = 3.0, L/N = 10, LBY = 8 and e = 2.0. For the parameters, LRF, L/N and LBY are the filtering criteria for network links, which can be used to remove excessive connections. The e value is another important parameter for filtering nodes. Finally, the keyword nodes and links that are selected according to the above criteria form a keyword co-occurrence network, which includes 777 nodes and 4043 network links, and the network density is 0.0134. In addition, according to the processing results of Citespace, the modularity value of the network is 0.5483, the weighted mean silhouette value is 0.7649 and the harmonic mean value is 0.6388. Here, the modularity and silhouette values are the measurement indicators describing the network structure and also the quality indicators evaluating the analyzing effect. These results reflect that the results of node clustering are reasonable, the community boundaries of clustering are significant enough and the homogeneity of clustering categories is low. Through further visualization, the keyword co-occurrence network in the timeline view is shown in Figure 3, which also shows the main clustering categories of nodes and the keyword evolution path under each category. The keywords in each category have a co-occurrence relationship with other keywords in the same cluster category, and also show a co-occurrence relationship with keywords in different cluster categories.

**Figure 4:**
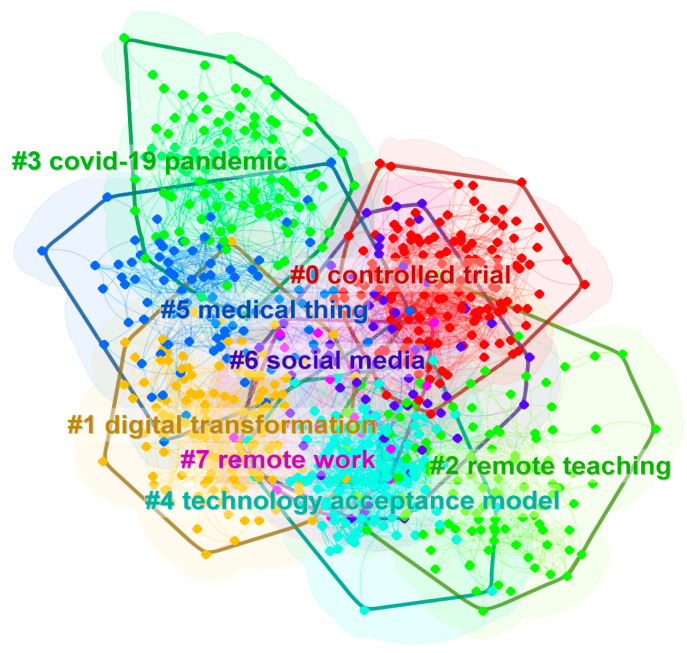
Clustering distribution of keyword co-occurrence network (cluster view). This figure corresponds to Figure 3 and omits the labels of all nodes, which highlights the clustering categories and the corresponding nodes. The color of the label indicates the category and the nodes that belong to a specific category present a boundary.

After switching to the cluster view, the co-occurrence relationship between nodes and the cluster community boundary can be seen in Figure 4. The clustering categories in this figure are consistent with those in Figure 3, which is another visual presentation. The nodes in the keyword co-occurrence network are divided into eight main categories.

Further, based on the document clustering analysis, the keyword information in the original data forms a keyword co-occurrence network as shown in Figure 4. Among the nodes, there are eight main cluster categories. There is a certain overlap of nodes between different categories, which reflects the potential semantic structure relationship in the research field. The labels of each category come from the article title. Citespace applies the LSI, LLR and MI algorithms to generate clusters. The list of labels for each category is shown in the following Table 4, which supports the interpretation of Figure 3 and Figure 4. The label keywords shown in the table can help to understand the meaning of specific clustering categories. In general, LLR is the best way to determine cluster category labels.

Table 4 shows that the largest category (Cluster #0) has 148 nodes, and the silhouette value is 0.745. The most prominent cluster labels generated by LLR, LSI and MI are, respectively, controlled trial, COVID-19 pandemic and 19-derived pandemic. The primary literature citing this category is the study by Dura Perez et al. (2022) [18]. This article pointed out that restrictive measures for epidemic prevention and control rely on the application of information and communication technology, but the impact of restrictive measures and technology application on the mental health of residents needs further exploration. The three keywords with the highest frequency in this category are health (331), care (224) and mental health (190).

The second largest category (Cluster #1) has 130 nodes, and the silhouette value is 0.686. The most prominent cluster labels generated by LLR, LSI and MI are digital transformation, COVID-19 pandemic and 19-derived pandemic, respectively. The most important literature citing this category is the work of Alam et al. (2021) [19] This article pointed out that a management information system that can automatically learn should be constructed to adapt to the trend of pandemic evolution and effectively curb the epidemic. The three keywords with the highest frequency in this category are impact (604), COVID-19 (449) and COVID-19 pandemic (378).

The third largest category (Cluster #2) has 114 nodes, and the silhouette value is 0.722. The most prominent cluster labels generated by LLR, LSI and MI are remote teaching, COVID-19 pandemic and 19-derived pandemic, respectively. The most important literature citing the category is the work of Basaran et al. (2022) [20], which pointed out that the development of COVID-19 has promoted the status of technology in the education process, but the impact of the pandemic on teachers’ technology perception is relatively limited. The three keywords with the highest frequency in this category are higher education (338), education (290) and virtual reality (245).

The fourth largest category (Cluster #3) has 108 nodes, and the silhouette value is 0.831. The most prominent cluster labels generated by LLR, LSI and MI are COVID-19 pandemic, artistic intelligence and 19-derived pandemic, respectively. The literature most closely related to this category is the work of Dong et al. (2021) [21] The mentioned research pointed out that building an intelligent community that can make full use of the perception ability and seamless connection characteristics based on the Internet of Things, incorporating the technology into the epidemic prevention and control system, is an urgent solution. The three keywords with the highest frequency in the category are artistic intelligence (811), deep learning (376) and machine learning (332).

The fifth largest category (Cluster #4) has 107 nodes, and the silhouette value is 0.814. The most prominent cluster labels generated by LLR, LSI and MI are technology acceptance model, COVID-19 pandemic and 19-derived pandemic, respectively. The literature most closely related to this category is the research of Adegoke et al. (2021) [22], which pointed out that the main factors for the real estate industry to adopt VR technology during the COVID-19 pandemic include performance expectations, effort expectations, social impacts, convenience conditions, hedonic motives and internal values. The three keywords with the highest frequency in the category are technology (685), model (542) and information technology (458).

The sixth largest category (Cluster #5) has 87 nodes, and the silhouette value is 0.803. The most prominent cluster labels generated by LLR, LSI and MI are medical thing, COVID-19 pandemic and 19-derived pandemic, respectively. The literature most closely related to this category is also the research of Dong et al. (2021) [21], which shows that Cluster # 5 and Cluster # 3 have potential connections. The three most frequent keywords in this category are system (360), internet (340) and challenge (211).

The seventh largest category (Cluster #6) has 61 nodes, and its silhouette value is 0.759. The most prominent cluster labels generated by LLR, LSI and MI are social media, COVID-19 pandemic and 19-derived pandemic, respectively. The literature most closely related to this category is the study of Patra et al. (2022) [23] The mentioned study discussed the evolution of fake news during COVID-19, and proposed implementation strategies to deal with information chaos in the digital era. The three keywords with the highest frequency in this category are information (297), social media (275) and communication (135).

The eighth largest category (Cluster #7) has 20 nodes, and the silhouette value is 0.906. The most prominent cluster labels generated by LLR, LSI and MI are remote work, COVID-19 pandemic and COVID-19 pandemic, respectively. The literature most closely related to the category is the work of Mansour et al. (2022) [24], which pointed out the importance of training digital technology applications for nursing work responding to the COVID-19 pandemic, but there are still some conceptual obstacles preventing staff from accepting new technologies. The three keywords with the highest frequency in this category are work (62), space (43) and resource (41).

#### 3.2.2. Literature Co-Citation Network Analysis

The analysis process of the literature co-citation network is similar to the process of the keyword co-occurrence network. Correspondingly, the network nodes are also divided into three 1-year time intervals. Table 5 shows the network screening criteria and the network attributes under each time interval. There are 265 articles from 2020 added to the analysis sample, and 795 network links are retained. From 2021, 516 articles and 1548 network links are selected. Moreover, from 2022 there are 441 articles corresponding to 1323 network links remaining.

**Figure 5:**
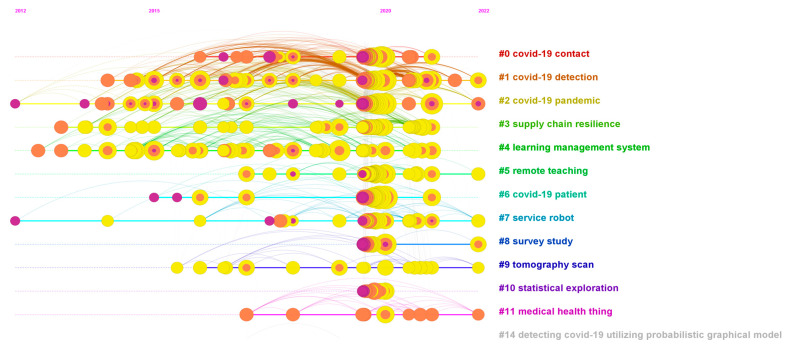
Literature co-citation network (timeline view). This figure omits the labels of all nodes avoid congestion, which highlights the network links. The colors of links correspond to the categories. The horizontal axis is the publication time, and the vertical axis also indicates the main clustering categories.

To further explore the knowledge flow process of literature, this paper uses the co-citation method to analyze the evolution trend of literature citation, thus obtaining the co-citation network of literature under the timeline view as shown in Figure 5. The network visualization reflects the co-citation relationship between core articles, with 772 nodes and 3244 network links, and a 0.0109 network density. In addition, the modularity value of the network is 0.5447, the weighted mean silhouette value is 0.8942 and the harmonic mean value is 0.677. In terms of the analysis process, co-citation network analysis and keyword co-occurrence analysis have much in common. First, the same network screening criteria are adopted, namely g-index (k = 25), LRF = 3.0, L/N = 10, LBY = 8 and e = 2.0. Second, the text of the clustering labels also comes from the title information of each article. These similarities help to find a connection between the two networks, which can provide a sufficient explanation for identifying key literature. Third, the keyword co-occurrence network shows the mutual relationship of topics, and the co-citation network presents the mutual relationship between literature nodes.

However, unlike the keyword co-occurrence network, the co-citation network reflects more on the knowledge structure evolution. As introduced in Section 2, the co-citation relationship suggests the knowledge base and research frontiers in a particular research field. The knowledge base is a collection of important articles identified by the citing behavior of frontier articles, which reflects a citation development trajectory. The research frontier is a collection of cited articles identified by the continuous development of frontier literature for a certain research branch. Therefore, knowledge base and research frontier are a pair of interrelated concepts. Through the theory, the literature co-citation network can identify some common references from the analyzed literature samples. These identified references constitute the knowledge base of the research field but do not necessarily appear in the analyzed samples. With the evolution of the knowledge field, some studies have received more attention and the corresponding citations will grow rapidly, which indicates frontier research. By focusing on the knowledge base and frontier research, we can quickly sort out the research context, and then determine the next research direction.

**Figure 6:**
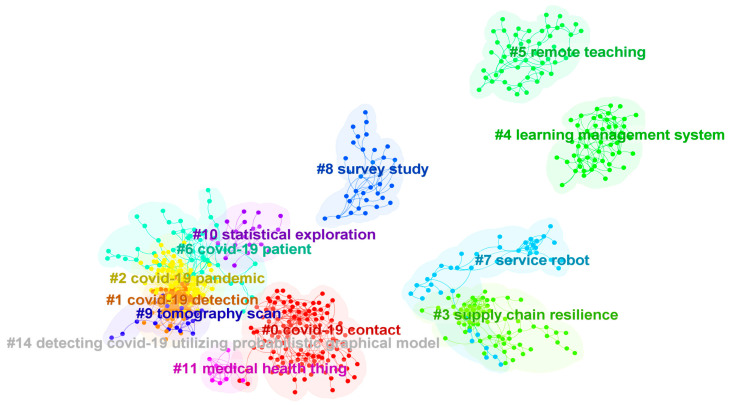
Literature co-citation network (cluster view). This figure corresponds to Figure 5 and omits the labels of all nodes, which aims to highlight the clustering categories. Every category label has a color, and the boundary of the category is presented.

Figure 6 is the literature co-citation network in the cluster view, which shows the distribution of literature nodes in each cluster category. The largest category (Cluster #0) has 123 nodes, and the silhouette value is 0.902. The most prominent cluster labels generated by LLR, LSI and MI are COVID-19 contact, COVID-19 pandemic and vital sign, respectively. The most prominent literature citing this category is the work of Islam et al. (2021) [25], which reviewed deep learning technology for COVID-19 diagnosis, and pointed out that the unstructured and non-standard data sets are the main challenges for the deep learning application. The most three frequently cited articles in this category are Ting et al. (2020) [26], Hollander et al. (2020) [27], Ferretti et al. (2020) [28].

The second largest category (Cluster #1) has 92 nodes, and the silhouette value is 0.828. The most prominent cluster labels generated by LLR and LSI are the same: COVID-19 detection. In addition, the first label generated by MI is vital sign. The most prominent literature citing this category is the work of Islam et al. (2021) [25], which is the same as Cluster # 0. The three most frequently cited articles in this category are Oztuk et al. (2020), Apostolopoulos et al. (2020) and He et al. (2016) [29,30,31].

The third largest category (Cluster #2) has 89 nodes, and the silhouette value is 0.717. The most prominent cluster labels generated by LLR, LSI and MI are COVID-19 pandemic, artificial intelligence and vital sign, respectively. The most prominent reference in this category is the work of Ozturk et al. (2020) [29], and the article proposed a method to automatically detect COVID-19 based on X-ray image technology. The three most frequently cited articles in this category are Ai et al. (2020), Li (2020a) et al. and Fang et al. (2020) [32,33,34].

The fourth largest category (Cluster #3) has 66 nodes, and the silhouette value is 0.984. The most prominent cluster labels generated by LLR, LSI and MI are supply chain resilience, COVID-19 pandemic and vital sign, respectively. The most prominent literature citing this category is the work of Queiroz et al. (2022) [35], which pointed out that the operation and coordination of supply chain resources are important for coping with the COVID-19 pandemic, and the digital twin method can provide a copy for the enterprise supply chain and improve the alertness level. The three most frequently cited articles in this category are Ivanov (2020), Dwivedi et al. (2020) and Bolin (2014) [36,37,38].

The fifth largest category (Cluster #4) has 53 nodes, and the silhouette value is 0.984. The most prominent cluster labels generated by LLR, LSI and MI are learning management system, COVID-19 pandemic and vital sign. The most prominent literature citing this category is the work of Akhter et al. (2022) [39], which indicated that technical support and organizational willingness are important factors affecting the online learning effect during the COVID-19 pandemic. The two most frequently cited articles in this category are Henseler et al. (2015) and Hair et al. (2019) [40,41].

The sixth largest category (Cluster #5) has 51 nodes, and the silhouette value is 0.932. The most prominent cluster labels generated by LLR, LSI and MI are remote teaching, COVID-19 pandemic and vital sign. The most prominent literature citing this category is the research of Meletiou-mavrotheris et al. (2022) [42] This mentioned research argued that under the influence of the COVID-19 pandemic, teaching quality and accessibility show a serious trend of inequality, and building a high-quality online learning platform for all learners is very important. The two most frequently cited papers in this category are Dhawan (2020) and Bao (2020) [43,44].

The seventh largest category (Cluster #6) has 50 nodes, and the silhouette value is 0.918. The most prominent cluster labels generated by LLR, LSI and MI are COVID-19 pandemic, COVID-19 patient and vital sign, respectively. The most prominent literature citing this category is the work of Mehrdad et al. (2021) [45], which showed that the use of wearable smart devices can help control the epidemic spread and track specific biomarkers by detecting virus infection symptoms and predicting the infection possibility. The three most frequently cited papers in this category are Huang et al. (2020), Zhu et al. (2020) and Guan et al. (2020) [46,47,48].

The eighth largest category (Cluster #7) has 42 nodes, and the silhouette value is 0.954. The most prominent cluster tags generated by LLR, LSI and MI are service robot, COVID-19 pandemic and vital sign. The most prominent literature citing this category is the work of Özekici et al. (2022) [49], which pointed out that the anxiety caused by COVID-19 significantly improved users’ acceptance of virtual reality, which provides implications for tourism restoration. The three most frequently cited articles in this category are Gössling et al. (2021), Yang et al. (2020) and Dontu et al. (2020) [50,51,52].

The ninth largest category (Cluster #8) has 35 nodes, and the silhouette value is 0.996. The most prominent cluster tags generated by LLR, LSI and MI are survey study, COVID-19 pandemic and vital sign. The most prominent literature citing this category is the work of Dubey et al. (2020) [53], which pointed out that the COVID-19 pandemic brought severe psychosocial problems with social event risks, and accelerating digital technology to develop plans to intervene in negative psychological conditions is necessary. The most frequently cited articles in this category are Brooks et al. (2020), Cao et al. (2020) and Holmes et al. (2020) [54,55,56].

The tenth largest category (Cluster #9) has 19 nodes, and the silhouette value is 0.844. The most prominent cluster labels generated by LLR, LSI and MI are, respectively, topography scan, COVID-19 detection and COVID-19 pandemic. The most prominent literature citing this category is the work of Suri et al. (2022) [57], and the research pointed out that the application of artificial intelligence technology in CT scanning can locate and segment COVID-19 focus faster and more reliably. The most frequently cited articles in this category are Selvaraju et al. (2017) and Horry et al. (2020) [58,59].

The eleventh largest category (Cluster #10) has 19 nodes, and the silhouette value is 0.938. The most prominent cluster labels generated by LLR, LSI and MI are COVID-19 pandemic, artificial intelligence and visual sign, respectively. The most prominent literature citing this category is the work of Chen et al. (2021) [60], which indicated that the application of artificial intelligence in combating the COVID-19 pandemic can seek breakthroughs of data resources, thus improving the application scope and potential. The most frequently cited articles in this category are Li (2020b) et al., van Doremalen et al. (2020) and Wu et al. (2020) [61,62,63].

The twelfth largest category (Cluster #11) has 10 nodes, and the silhouette value is 0.981. The most prominent cluster labels generated by LLR, LSI and MI are medical health thing, artificial intelligence and COVID-19 pandemic, respectively. The most prominent literature citing this category is the study of Madhavan et al. (2021) [64], which suggested that it is easy to obtain false positive and false negative results with polymerase chain reaction and reverse transcription polymerase chain reaction, thus using CT or X-ray based on artificial intelligence can become an effective alternative to identify patients with COVID-19. The most frequently cited articles in this category are Hossain et al. (2020), Laguarta et al. (2020) and Hossain (2017) [65,66,67].

The thirteenth largest category (Cluster #14) has two nodes, and the silhouette value is 1. The most prominent cluster labels generated by LLR, LSI and MI are detecting COVID-19 utilizing a probabilistic graphical model, artificial intelligence and COVID-19 pandemic, respectively. The most prominent literature cited in this category is the work of Rahman et al. (2021) [68], which stated that the application of artificial intelligence in the prevention and control of COVID-19 is facing challenges, such as lack of resource support, and legal and ethical risks. The most frequently cited articles in this category are Jiang et al. (2017) and Davenport et al. (2019) [69,70] Finally, the main text labels in each category are shown in Table 6.

#### 3.2.3. Core Literature Identification

As shown in Table 7, although the keyword co-occurrence network divides keywords into eight main categories, the silhouette value of each category is relatively low, and only the silhouette value of Cluster #7 is higher than 0.9, which indicates that there is a certain topic overlap between various categories. Therefore, on the basis of text mining, the above keyword clustering categories have the potential for integration, thus forming a new category. These keywords provided hints to find the core articles and the analysis process was finished in Citespace. Next, this study used the keywords to conduct a discussion that presents the research evolution. From the analysis results of keywords, the clustering categories mainly reflect three new kinds of topics. The first new category is mainly about public health and medical care, including Cluster #0 and Cluster #1. The second new category is mainly about education, labor, social media and other aspects of social governance, including Cluster #2, Cluster #6 and Cluster #7. The third new category is mainly about artificial intelligence, information technology, the Internet and other emerging technologies, including Cluster #3, Cluster #4 and Cluster #5. Moreover, the distribution of these topics shows that epidemic prevention and control is a systematic project, which requires the cooperation of multiple departments to implement the virus containment strategy.

Table 8 shows the representative articles in the cluster analysis of the literature co-citation network. Similar to the keyword co-occurrence network, the clustering categories of the literature co-citation network also reflect three new types of topics. The first new category is digital technology applications related to COVID-19 diagnosis, including Cluster #0, Cluster #9, Cluster #11 and Cluster #14. The second new category is about other applications of digital technology in the medical process of the COVID-19 pandemic, including Cluster #1, Cluster #2, Cluster #6 and Cluster #10. The third new category is the strategy of social governance and digital transformation of the industrial economy in response to the challenges of the COVID-19 pandemic, including Cluster #3, Cluster #4, Cluster #5, Cluster #7 and Cluster #8. In addition, Cluster #3, Cluster #7 and Cluster #8 are mainly related to the digital transformation of social governance, while Cluster #4 and Cluster #5 are mainly related to the digital transformation of education. The distribution of the above categories further shows that the process of digitalization of epidemic prevention and control needs to be promoted simultaneously from both technical and social governance aspects. The R&D and application of digital technology need to be planned in combination with the needs of social governance, which is based on the premise of effectively containing the epidemic spread.

**Figure 7:**
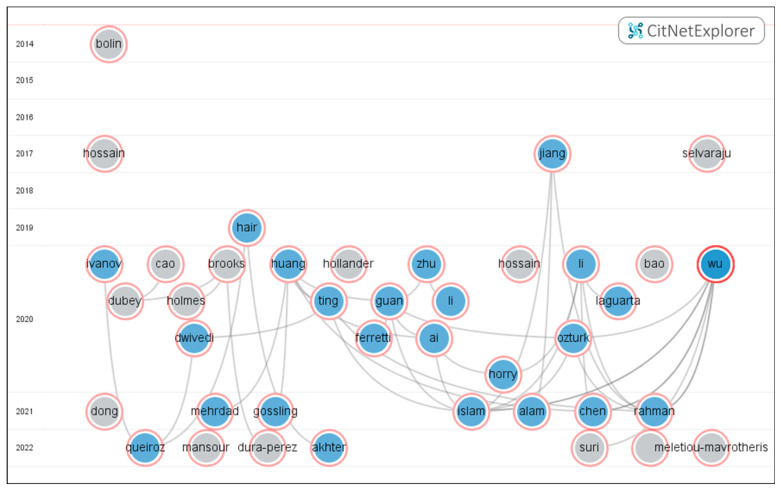
Core literature citation network based on CitNetExplorer. Representative articles [18,19,20,21,24,25,26,27,28,29,30,32,33,34,35,36,37,38,39,41,42,44,45,46,47,48,50,51,52,53,54,55,56,57,58,59,60,61,62,63,65,66,67,68,69] were included and the articles [22,23,49,64] were not included. The left axis of the figure shows the publishing year of the nodes, which are labeled with the name of the first author. The link reflects that two literature nodes have a citation relationship.

This study applied CitNetExplorer to analyze the citation relationships between 49 core articles, and the results are shown in Figure 7. In addition to the timespan 2020–2022, some of the core documents are from 2019 or before 2019. It should be noted that four of the core articles are published online, and cannot be identified by CitNetExplorer and are not shown in the above results. Therefore, 43 citation network links have been formed among the 45 articles analyzed, and 16 articles of the 45 have citation relations in the figure, while the other 29 articles present isolated characteristics. The most-cited article is Huang et al. (2020) [46], which has been cited six times. The following ones are Li (2020), Guan (2020) and Wu (2020) [33,48,63], which were cited five times, four times and four times, respectively. The citation relationship shown in Figure 7 shows that there are still close citation relationships within the limited literature scope, which constitutes an important knowledge base in the field of digital epidemic prevention and control research. While other isolated nodes do not form a reference relationship in the figure, these articles have potential topic coupling relationships and reflect different digitalization directions.

**Figure 8:**
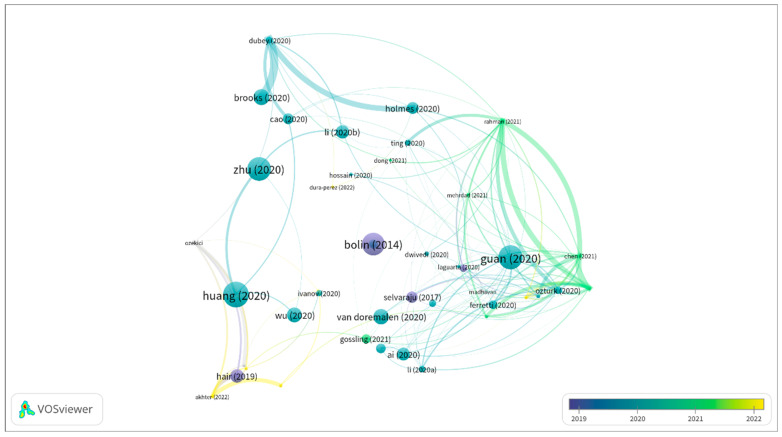
Bibliographic coupling analysis of core articles based on VOSviewer. Representative articles [18,19,20,21,22,23,24,25,26,27,28,29,30,32,33,34,35,36,37,38,39,41,42,44,45,46,47,48,49,50,51,52,53,54,55,56,57,58,59,60,61,62,63,64,65,66,67,68,69] were included. Different colors in the figure represent the publishing years of literature nodes, and the thickness of the link indicates the coupling relationship between the nodes, which means that a thicker link reflects that these two articles have more mutual references. Node size means the citation frequency and also the weight in the network.

As shown in Figure 8, VOSviewer is used to conduct a bibliographic coupling analysis of 49 identified core articles. The analysis results show five clustering categories, and the fifth category is a combining category that includes all the isolated nodes. 42 of the 49 articles have literature coupling relations. The article of Islam et al. (2021) [25] has the highest strength of coupling relations, with a total link strength of 41, followed by Dubey et al. (2020), Chen et al. (2021) and Rahman (2021) [53,60,68], and the corresponding total link strengths are 33.33, 39 and 41, respectively.

Through the above bibliometric analysis, we can conclude some common trends. For example, the COVID-19 pandemic is obviously one of the core contents, and digital transformation takes a certain proportion but is not obvious, which reflects that the digital process of epidemic prevention and control is still in the development stage. In addition, the results of the bibliometric analysis also reflect the characteristics of differentiation. In the clustering results obtained with the LLR algorithm, there are many differences in the main categories under various dimensions of literature citation, while the main cluster categories obtained with the MI and LSI algorithms are less different. This result reflects the fact that the LLR algorithm can more effectively explore the topic distribution of literature, while the MI and LSI algorithms are more suitable for testing the consistency of results. Therefore, the core articles can be divided into the following branches for literature review and discussion.

## 4. Discussion

### 4.1. Technological Embedding of Artificial Intelligence

The literature in this category mainly introduced the specific routes of embedding artificial intelligence technology into the epidemic prevention and control process, and includes 15 articles. Selvaraju et al. (2017) [58] and Jiang et al. (2017) [69] are the earlier articles of the knowledge base, pointing out the key contents of data availability and interpretability of results for artificial intelligence. The research published after the outbreak of COVID-19 in 2020 can be divided into several sub-topics. The first one is the application of new artificial intelligence technology to replace traditional diagnostic protocols [30,59,66]. The second aspect is the application of epidemic prevention and control [26,37]. The third one is potential technical risk prevention [65]. Studies in 2021 and later showed a series of evolutions in the above three themes. First, the intelligent and automatic role of technology was more prominent in the diagnosis process, and more accurate analysis results were obtained [19,57,64]. Second, the application requirements for epidemic prevention and control were more clearly defined, such as perception ability and seamless features [21]. Finally, the requirements for data sample quality were further improved, pointing out the seriousness of the lack of data quality and legal and ethical risks [25,60,68].

### 4.2. Big Data Mining and Digital Twin

The literature in this category mainly introduced decision-making support for epidemic prevention and control and social governance through big data analysis and mining results, and includes nine articles. Hair et al. (2019) proposed an improved method for the application and interpretation of partial least square–structural equation modelling (PLS-SEM) analysis results [41], which is a knowledge base literature on research methods. This method had been used in many studies on epidemic prevention and control, indicating that public investigation is an important means to understand the epidemic situation. After the outbreak of COVID-19, related literature received less attention, mainly focusing on supply chain stability and industrial economic recovery [22,36]. With the epidemic, the attention on economic recovery and social restructuring is increasing rapidly, which can be divided into two categories. One is the promotion policy of online education [20,39,42]. The other one is the digital transformation of traditional industries and the system construction of digital twin [24,35,49].

### 4.3. Coordination of Epidemic Prevention and Control

The literature in this category mainly introduced the transformation and coordination process of epidemic prevention and control with the help of digital technology, which has a certain content connection with the first category, and includes nine articles. The category has no articles published before the outbreak; they are mainly distributed in the 2020–2021 timespan. According to the time sequence, the research topics of this category have obvious evolution characteristics. First, the research in 2020 mainly focused on epidemic prevention and control strategies, and the nature of the novel coronavirus makes digital technology show advantages [48,62]. Thus, applying digital technology to the stages of patient diagnosis [29,32,33,34], epidemiological investigation and epidemic development surveillance [28,45] can help to transform the traditional epidemic prevention approach. Further, the planning of epidemic prevention and control needs to be integrated with economic development. Despite the impact on the global industrial economy, the epidemic also bought in an opportunity to reshape the industrial development system [50].

### 4.4. Digital Social Governance in the Post-Pandemic Era

The literature in this category mainly introduced the role of digital technology and the development path of social governance in the pandemic era, and includes nine articles. Due to the transmission characteristics of COVID-19 [47,61,63], high intensity containment and control measures need to be considered. However, the public is faced with greater psychological pressure in the process of epidemic containment, which is prone to psychological health risks [54,55,56]. The above situation shows the necessity of using digital technology to collect patient data [46] and strengthen psychological ties [53], but the other aspect also reflects new mental health risks for the new technology [18]. In general, this category does not introduce much about digital technology, but suggests the need to accelerate the research on the digital technology application in social governance.

### 4.5. Risk Prevention in Digital Transformation

The literature in this category mainly introduced the possible risks and prevention strategies for the digital transformation in epidemic prevention and control, and includes seven articles. Two articles published before 2020 introduced data processing and analysis methods [38] and data acquisition methods using sensing devices [67], which have been widely used in the following investigation and research on the novel coronavirus pandemic. After the outbreak in 2020, the impact of the epidemic on various aspects of a region has received more attention, such as the impact on the education system [44], the impact on the commercial system [52] and the multiple impacts on the manufacturing industry and the economy [51]. In addition, the application of digital technology could also create a series of challenges, such as the false news prevalence on social media [23] and the financial burden and travel restrictions brought by telemedicine [27].

## 5. Conclusions

Through bibliometric analysis of prior literature, this paper identified some core articles on the research topic of digital transformation in COVID-19 prevention and control, and summarized the specific construction path of the open innovation mechanism for the digital technology application based on a systematic review, which provides certain support for a region’s strategy and policy formulation for epidemic prevention. Furthermore, this study systematically explored the ways in which digital technology supports COVID-19 prevention. Digital transformation seems to bring everything and everyone together, which forms an open innovation mechanism for the technology development of public health governance. A large number of studies around the topic of COVID-19 have been conducted since 2020, which were scattered in different research directions and research fields.

However, there are a lot of potential links among the prior studies. The bibliometrics analysis result showed complex citation relationships and presented an aggregation trend in various fields, reflecting the systematic knowledge structure of the above-mentioned hot topics. From the fragmented literature data, this paper focused on parts of key articles and explored the evolutionary route of digital transformation in the process of epidemic prevention and control. As indicated by the prior studies [71,72], open innovation is dynamic and evolutionary, which suggests that epidemic prevention planning should be continually updated. The findings showed that a variety of digital technology applications have been proposed, which are expected to be integrated into a policy framework characterized by an open innovation system, thus providing digital management implications for countries or regions under different levels of the epidemic outbreak as follows.

First, epidemic prevention policy needs to promote more digital technology innovation. The deep integration of digital technologies plays an important role in all aspects of epidemic prevention and control, which is necessary to contain the spread of COVID-19 and prevent more serious outbreaks in the future. However, it should be noted that digital technology related to epidemic prevention and control is dynamic, and virus mutation can prompt rapid adaptation of digital technology. Second, policy-makers can consider establishing an open innovation system for epidemic prevention and control. This system connects health, education, labor and transportation aspects based on digital transformation. Furthermore, the combination of the pandemic and digital transformation is blurring the boundaries of traditional sectors. Effective control of the epidemic increasingly requires collaboration between different sectors, as well as participation and support from the public. Third, institutions need to propose digital transformation planning for epidemic risk prevention. The process of digital transformation is changing as the pandemic evolves, which reshapes new social structures in the post-pandemic era. Compared with previous outbreaks, the transmission of the novel coronavirus is more hidden, more diffuse, and it has a longer incubation period, which strongly challenges the traditional prevention methods. The introduction of digital technology can make up for certain deficiencies, but the application of emerging technologies will also bring potential risks, such as personal information infringement and data security.

Due to the available data, the analysis of this study is mainly based on the established epidemic response plan. Therefore, the study has a certain lag compared to the development trend of the latest COVID-19 pandemic. However, the analytical framework of digital transformation proposed in this paper can be used as a research basis for future studies, and some adjustments can be made based on the latest situation of the epidemic. Moreover, further retrieval of preprint literature data and literature analysis for the five topics identified in this paper are possible to reduce the impact of publication bias.

## Figures and Tables

**Table 1:** The systematic literature analysis process.

Analysis Steps	Analytical Dimensions	Dimension Description
Step 1: Overall distribution analysis	(1) Basic bibliometric distribution	Identify the dynamic development trend and main distribution elements through the literature statistics from the perspectives of time, journals, authors and institutions
	(2) Research topic distribution	Identify the main literature topics with large distribution through the literature statistics from the perspectives of research areas, WOS categories and citation topics meso
	(3) Research geography and foundation	Identify the main sources of element distribution through the literature statistics from the perspectives of countries/regions and foundations
Step 2: Literature network structure analysis	(1) Keyword co-occurrence network analysis	Extract keywords from document texts and construct a network according to two keywords’ relationship in the same document, thus exploring the semantic structure in specific research fields
	(2) Literature co-citation network analysis	Build a literature co-citation network based on the commonly cited literature, and analyze the dynamic evolution trend in the research field through network attributes
	(3) Core literature identification	Based on the analysis results of the keyword co-occurrence network and literature co-citation network, select a batch of core literature according to the network attributes
Step 3: Content analysis of core literature	Specific categories of core literature	Divide the core literature into several categories, and then analyze and discuss each category in combination with the literature content

**Table 2:** Top 10 classifications and the corresponding article numbers.

Rank	Research Areas	Number	WOS Categories	Number	Citation Topics Meso	Number
1	Computer Science	1789	Education Educational Research	1134	6.11 Education & Educational Research	1004
2	Engineering	1360	Computer Science Information Systems	1014	6.3 Management	955
3	Education Educational Research	1345	Public Environmental Occupational Health	796	1.104 Virology-General	891
4	Business Economics	1059	Environmental Sciences	768	6.185 Communication	493
5	Science Technology Other Topics	849	Health Care Sciences Services	719	1.273 Health Literacy & Telemedicine	492
6	Environmental Sciences Ecology	848	Engineering Electrical Electronic	698	4.187 Security Systems	392
7	Public Environmental Occupational Health	796	Medical Informatics	536	4.17 Computer Vision & Graphics	304
8	Health Care Sciences Services	790	Management	501	4.13 Telecommunications	299
9	Medical Informatics	536	Environmental Studies	466	1.14 Nursing	241
10	Telecommunications	429	Green Sustainable Science Technology	459	1.44 Nutrition & Dietetics	193

**Table 3:** Keyword co-occurrence network attributes in each year.

1-Year Slices	Criteria	Space	Nodes	Links/All
2020	g = 36, k = 25	4442	272	816/3437
2021	g = 86, k = 25	12,512	586	1758/17,473
2022	g = 89, k = 25	12,961	599	1797/18,503

Note: In the criteria, g refers to a g-index value that presents a criterion for literature selection, and k is a parameter in the g-index, which indicates the scaling factor.

**Table 4:** The label list of clustering categories in the keyword co-occurrence network.

Cluster	Size	Silhouette	Year	Label (LSI)	Label (LLR)	Label (MI)
0	148	0.745	2020	COVID-19 pandemic; mental health; controlled trial; study protocol; COVID-19 era|empirical evidence; New York city; health care; early stage; health behavior	controlled trial; mental health; study protocol; physical activity; social isolation	19-derived pandemic; secondary education te
1	130	0.686	2021	COVID-19 pandemic; digital transformation; digital technologies; case study; COVID-19 era|online learning; technology acceptance model; university student; virtual reality; distance learning	digital transformation; supply chain resilience; supply chain disruption; medium-sized enterprises; dynamic capabilities	19-derived pandemic; secondary education te
2	114	0.722	2020	COVID-19 pandemic; case study; remote teaching; distance learning; online learning|technology acceptance model; behavioral intention; learning management system; actual use; continuance intention	remote teaching; distance learning; digital competence; distance education; online teaching	19-derived pandemic; secondary education te
3	108	0.831	2020	artificial intelligence; COVID-19 patient; COVID-19 detection; COVID-19 pandemic; deep learning|COVID-19 classification; retrospective study; COVID-19 outbreak; automatic detection; CT-based COVID-19 diagnosis	COVID-19 pandemic; COVID-19 detection; COVID-19 patient; COVID-19 pneumonia; COVID-19 diagnosis	19-derived pandemic; secondary education te
4	107	0.814	2020	COVID-19 pandemic; technology acceptance model; empirical study; online learning; behavioral intention|online therapy; online purchasing; banking sector; using food delivery app; clinical practice	technology acceptance model; moderating effect; learning management system; actual use; behavioral intention	19-derived pandemic; secondary education te
5	87	0.803	2020	COVID-19 pandemic; medical thing; artificial intelligence; COVID-19 outbreak; COVID-19 era|food supply chain; behavioral intention; communication technology; using blockchain technology; anonymous IoT-based e-health monitoring system	medical thing; fog computing; COVID-19 pandemic; smart healthcare; cloud computing	19-derived pandemic; secondary education te
6	61	0.759	2020	COVID-19 pandemic; social media; fake news; social network; case study|public perception; digital literacy; digital transformation; empirical evidence; posttraumatic stress disorder	social media; fake news; social network; COVID-19 vaccine; social media usage	19-derived pandemic; secondary education te
7	20	0.906	2021	COVID-19 pandemic; remote work; employee engagement; COVID-19 context; healthcare organization|work-related well-being; integrated perspective; jd-r theory; technology acceptance model; travel behavior	remote work; employee engagement; healthcare organization; crafting new technology; corporate well-being programme	COVID-19 pandemic; 19-derived pandemic

**Table 5:** Literature co-citation network attributes in each year.

1-Year Slices	Criteria	Space	Nodes	Links/All
2020	g = 29, k = 25	36,913	265	795/6487
2021	g = 53, k = 25	127,927	516	1548/24,647
2022	g = 43, k = 25	134,581	441	1323/13,073

**Table 6:** Label list of clustering categories in literature co-citation network.

Cluster	Size	Silhouette	Year	Label (LSI)	Label (LLR)	Label (MI)
0	123	0.902	2019	COVID-19 pandemic; COVID-19 contact; artificial intelligence; tracing app; big data|machine learning; digital contact-tracing; COVID-19 cases; comparative study; Saudi Arabia	COVID-19 contact; tracing app; COVID-19 pandemic; IoT-enabled framework; COVID-19 tracing app	vital sign; methods prospect; using
1	92	0.828	2019	COVID-19 detection; COVID-19 patient; deep learning; COVID-19 diagnosis; chest x-ray|artificial intelligence; COVID-19 pneumonia; COVID-19 pandemic; chest CT; chest radiograph	COVID-19 detection; COVID-19 pandemic; deep learning; COVID-19 diagnosis; chest x-ray	vital sign; methods prospect; using
2	89	0.717	2019	artificial intelligence; COVID-19 patient; COVID-19 pneumonia; COVID-19 pandemic; COVID-19 diagnosis|COVID-19 severity; prospective validation; clinical characteristics; novel coronavirus; cov-2 infection	COVID-19 pandemic; COVID-19 pneumonia; COVID-19 diagnosis; chest CT scan; chest CT	vital sign; methods prospect; using
3	66	0.984	2019	COVID-19 pandemic; digital transformation; supply chain resilience; digital technologies; COVID-19 crisis|supply chain management; mediating role; digital supply chain management; supply chain analytics; COVID-19 disruption	supply chain resilience; digital transformation; supply chain management; firm resilience; enhancing supply chain resilience	vital sign; methods prospect; using
4	53	0.984	2017	COVID-19 pandemic; education institution; empirical study; learning management system; online learning|remote teaching; online teaching; digital transformation; digital technologies; distance learning	learning management system; actual use; structural equation modeling approach; mobile learning acceptance; mediation effect	vital sign; methods prospect; using
5	51	0.932	2020	COVID-19 pandemic; remote teaching; online teaching; online learning; case study|experience case; university education resilience; religious education; pandemic hybrid teaching; remote digital learning	remote teaching; online teaching; academic performance; COVID-19 pandemic; online education	vital sign; methods prospect; using
6	50	0.918	2019	COVID-19 patient; artificial intelligence; COVID-19 pandemic; machine learning; COVID-19 pneumonia|deep learning technique; smartphone technology; artificial intelligence application; vaccination rollout management; applying blockchain	COVID-19 pandemic; COVID-19 patient; prospective validation; clinical characteristics; machine learning model	vital sign; methods prospect; using
7	42	0.954	2019	COVID-19 pandemic; virtual reality; COVID-19 era; service robot; virtual reality tourism|dynamic capabilities; exploratory study; digital technology; generalized belief; problematic smartphone use	service robot; virtual reality tourism; virtual tourism; hospitality service; virtual reality	vital sign; methods prospect; using co
8	35	0.996	2020	COVID-19 pandemic; COVID-19 lockdown; virtual reality; digital divide; survey study|qualitative study; observational study; technology acceptance model; SARS experience; big data	survey study; COVID-19 pandemic; psychological distress; COVID-19 lockdown; social networking site	vital sign; methods prospect; using
9	19	0.844	2019	COVID-19 detection; tomography scan; COVID-19 classification; COVID-19 lung; deep learning|efficient deep neural network framework; COVID-19 lung infection segmentation; artificial intelligence paradigm; using unseen deep learning; varying glass ground opacities	tomography scan; COVID-19 lung; COVID-19 classification; advances challenge; privacy-aware method	COVID-19 pandemic; using conference tool
10	19	0.938	2020	artificial intelligence; deep learning; COVID-19 pandemic; coronavirus disease; statistical exploration|COVID-19 using regression analysis; death rate; COVID-19 detection; data-driven epidemic intelligence strategies; tracing technologies	COVID-19 pandemic; statistical exploration; COVID-19 dataset; small molecule; cov-2 discovery	vital sign; methods prospect; using
11	10	0.981	2020	artificial intelligence technique; medical thing; COVID-19 framework; using transfer learning; medical health thing|auxiliary diagnosis; COVID-19 pandemic; 5g-enabled federated learning; privacy-enhanced data fusion; COVID-19 application	medical health thing; COVID-19 framework; COVID-19 patient monitoring; ANN assisted-iot; smart ontology-based iot framework	COVID-19 pandemic; artificial intelligence
14	2	1	2018	artificial intelligence; detecting COVID-19 utilizing probabilistic graphical model; drug discovery; bibliometric analysis; 21st century age|learning approach; outcome severity; knowledge extraction; COVID-19 clinical status associate; unsupervised machine	detecting COVID-19 utilizing probabilistic graphical model; drug discovery; 21st century age; machine learning approaches; artificial intelligence	COVID-19 pandemic; artificial intelligence

**Table 7:** Representative keywords from keyword co-occurrence network.

Cluster	Keywords	Year	Freq	Degree	Centrality
0	health	2020	331	6	0
	care	2020	224	11	0
	mental health	2020	190	21	0.01
1	impact	2020	604	18	0.01
	COVID-19	2020	449	1	0
	COVID-19 pandemic	2020	378	5	0
2	higher education	2020	338	20	0.01
	education	2020	290	12	0
	virtual reality	2020	245	12	0.01
3	artificial intelligence	2020	811	16	0
	deep learning	2020	376	22	0.01
	machine learning	2020	332	13	0.01
4	technology	2020	685	22	0.02
	model	2020	542	21	0.02
	information technology	2020	458	34	0.01
5	system	2020	360	11	0.01
	internet	2020	340	20	0.02
	challenge	2020	211	14	0
6	information	2020	297	15	0.01
	social media	2020	275	9	0
	communication	2020	135	6	0
7	work	2020	62	19	0.02
	space	2020	43	7	0
	resource	2021	41	6	0

**Table 8:** Representative articles from the literature co-citation network.

Cluster	Article	Source	Year	Freq	Degree	Centrality
0	Ting DSW (2020) [26]	NAT MED	2020	145	9	0.09
	Hollander JE (2020) [27]	NEW ENGL J MED	2020	128	10	0.03
	Ferretti L (2020) [28]	SCIENCE	2020	119	17	0.02
1	Ozturk T (2020) [29]	COMPUT BIOL MED	2020	178	80	0.02
	Apostolopoulos ID (2020) [30]	PHYS ENG SCI MED	2020	160	87	0.03
	He KM (2016) [31]	PROC CVPR IEEE	2016	158	46	0.01
2	Ai T (2020) [32]	RADIOLOGY	2020	179	66	0.02
	Li L (2020) [33]	RADIOLOGY	2020	158	60	0.03
	Fang YC (2020) [34]	RADIOLOGY	2020	120	46	0.01
3	Ivanov D (2020) [36]	TRANSPORT RES E-LOG	2020	62	17	0
	Dwivedi YK (2020) [37]	INT J INFORM MANAGE	2020	57	2	0.03
	Bolin JH (2014) [38]	J EDUC MEAS	2014	50	4	0
4	Henseler J (2015) [40]	J ACAD MARKET SCI	2015	157	14	0.16
	Hair JF (2019) [41]	EUR BUS REV	2019	103	9	0.01
5	Dhawan Shivangi (2020) [43]	J EDU TECH SYS	2020	97	5	0.13
	Bao W (2020) [44]	HUM BEHAV EMERG TECH	2020	72	2	0
6	Huang CL (2020) [46]	LANCET	2020	203	11	0.02
	Zhu N (2020) [47]	NEW ENGL J MED	2020	151	3	0
	Guan W (2020) [48]	NEW ENGL J MED	2020	135	5	0.01
7	Gössling S (2021) [50]	J SUSTAIN TOUR	2021	66	8	0.01
	Yang GZ (2020) [51]	SCI ROBOT	2020	64	3	0
	Donthu N (2020) [52]	J BUS RES	2020	48	4	0.04
8	Brooks SK (2020) [54]	LANCET	2020	139	7	0.04
	Cao WJ (2020) [55]	PSYCHIAT RES	2020	64	6	0.16
	Holmes EA (2020) [56]	LANCET PSYCHIAT	2020	59	5	0.03
9	Selvaraju RR (2017) [58]	IEEE I CONF COMP VIS	2017	27	7	0
	Horry MJ (2020) [59]	IEEE ACCESS	2020	19	6	0
10	Li Q (2020) [61]	NEW ENGL J MED	2020	69	3	0
	van Doremalen N (2020) [62]	NEW ENGL J MED	2020	69	3	0
	Wu F (2020) [63]	NATURE	2020	67	12	0.02
11	Hossain MS (2020) [65]	IEEE NETWORK	2020	44	9	0.02
	Laguarta J (2020) [66]	IEEE OPEN J ENG MED	2020	16	4	0
	Hossain MS (2017) [67]	IEEE SYST J	2017	16	8	0
14	Jiang F (2017) [69]	STROKE VASC NEUROL	2017	19	2	0
	Davenport T (2019) [70]	FUTURE HEALTHC J	2019	13	1	0

## Data Availability

Data used for this review are publicly available at: https://www.webofscience.com (accessed on 1 July 2022). The updated data can be obtained by using the search strategy in this study.
